# Impact of an Educational Intervention on Women's Knowledge and Acceptability of Human Papillomavirus Self-Sampling: A Randomized Controlled Trial in Cameroon

**DOI:** 10.1371/journal.pone.0109788

**Published:** 2014-10-15

**Authors:** Gaëtan Sossauer, Michel Zbinden, Pierre-Marie Tebeu, Gisèle K. Fosso, Sarah Untiet, Pierre Vassilakos, Patrick Petignat

**Affiliations:** 1 Faculty of Medicine, University of Geneva, Geneva, Switzerland; 2 Department of Gynecology and Obstetrics, University Center Hospital, Yaoundé, Cameroon; 3 Department of Gynecology and Obstetrics, Gynecologic Division, Geneva University Hospitals, Geneva, Switzerland; 4 Geneva Foundation for Medical Education and Research, Geneva, Switzerland; University of New South Wales, Australia

## Abstract

**Objective:**

Human papillomavirus (HPV) self-sampling (Self-HPV) may be used as a primary cervical cancer screening method in a low resource setting. Our aim was to evaluate whether an educational intervention would improve women's knowledge and confidence in the Self-HPV method.

**Method:**

Women aged between 25 and 65 years old, eligible for cervical cancer screening, were randomly chosen to receive standard information (control group) or standard information followed by educational intervention (interventional group). Standard information included explanations about what the test detects (HPV), the link between HPV and cervical cancer and how to perform HPV self-sampling. The educational intervention consisted of a culturally tailored video about HPV, cervical cancer, Self-HPV and its relevancy as a screening test. All participants completed a questionnaire that assessed sociodemographic data, women's knowledge about cervical cancer and acceptability of Self-HPV.

**Results:**

A total of 302 women were enrolled in 4 health care centers in Yaoundé and the surrounding countryside. 301 women (149 in the “control group” and 152 in the “intervention group”) completed the full process and were included into the analysis. Participants who received the educational intervention had a significantly higher knowledge about HPV and cervical cancer than the control group (p<0.05), but no significant difference on Self-HPV acceptability and confidence in the method was noticed between the two groups.

**Conclusion:**

Educational intervention promotes an increase in knowledge about HPV and cervical cancer. Further investigation should be conducted to determine if this intervention can be sustained beyond the short term and influences screening behavior.

**Trials Registration:**

International Standard Randomised Controlled Trial Number (ISRCTN) Register ISRCTN78123709

## Introduction

In developed countries, cervical cancer screening with Pap smear and treatment of precancerous lesions has led to an important reduction of invasive cervical cancer incidence and mortality [1]–[3]. In developing countries, where basic health care services are either lacking or inaccessible and where there are significant barriers for preventive care implementation, cervical cancer is one of the leading causes of cancer death [4]. Because cervical cancer screening with Pap smear in developing countries is generally inefficient, alternative methods have been proposed [5]–[7], including visual inspection with acetic acid (VIA), visual inspection with Lugol's iodine (VILI) and human papillomavirus (HPV) self-sampling (Self-HPV).

VIA and VILI are attractive screening tests for low-resource countries, because they give an immediate result and allow treatment of abnormal lesions during the same medical visit in a “screen-and-treat” approach [8]. However, the method has important limitations such as highly variable sensitivity and specificity, which are very dependent on the examiner's expertise [9]. In this context, HPV testing may be an option for primary cervical cancer screening, as it is more sensitive and less dependent on the subjectivity and the training of health care professionals [10]. Moreover, it offers the option of being performed by women themselves with similar results compared to those performed by physicians [11]. As HPV testing only determines a potentially carcinogenic infection but no actual cervical lesion, VIA could be evaluated as an option to triage HPV positive women.

Prior to introducing a new screening method, it should be determined if the test is acceptable for the target population. This requires women's understanding of the role of HPV infection and its link with cervical cancer. Previous studies conducted in sub-Saharan African countries report a low awareness of HPV infection and cervical cancer indicating the need to inform the population [12]. The population should also be confident in the effectiveness and safety of the test. A study conducted in Uganda demonstrates that women want to perform Self-HPV, however there is no data to show whether women are confident in the test [13]. A Self-HPV study conducted in Cameroon, points out that women are less confident in their ability to collect and perform a self-test and trust their own results less than those of a HPV test performed by a physician [14].

To date, very few studies describe the effects of educational interventions on cancer prevention in sub-Saharan Africa [15]–[18]. Most of the reported trials evaluate oral and written information, e.g. printed leaflets, about cancer prevention and treatment. We hypothesize that the addition of a short and simple educational intervention through the use of a culturally-tailored video would improve knowledge and confidence in Self-HPV. Therefore, we have designed a simple randomized trial to assess the impact of an educational intervention on women's knowledge and confidence regarding Self-HPV.

## Materials and Methods

The protocol for this trial and supporting CONSORT checklist are available as supporting information; see Checklist S1 and Protocol S1.

### Determination of sample size

The required sample size (182 participants) was calculated for a comparison of proportions with an α of 0.05 and a β of 0.20, assuming that a difference of at least 20% for the primary outcome between interventional group and control would be scientifically interesting. The thus determined sample size was 182 participants. As we experienced high dropout rates in our previous studies in Cameroon and to answer the on-site demand of inclusion we recruited 66% more participants than calculated as at least necessary.

### Recruitment of participants

During July and August 2012, we recruited women aged between 25 and 65 years in 4 health care centers in Yaoundé and the surrounding countryside. Our visits to the health care centers were prepared by the local staff by word-of-mouth advertising and the suspension of informational posters indicating the dates of our visits, eligibility criteria and content of the study. The information clarified as well that the participation in the study with screening test and further investigations and treatment, if necessary, was free of charge for the participants. Interested women were invited to present themselves on the days of sampling.

Exclusion criteria included current menstruation, current pregnancy, previous hysterectomy or cervical surgery. A written informed consent was obtained from all participants.

### Randomization

**Figure 1:**
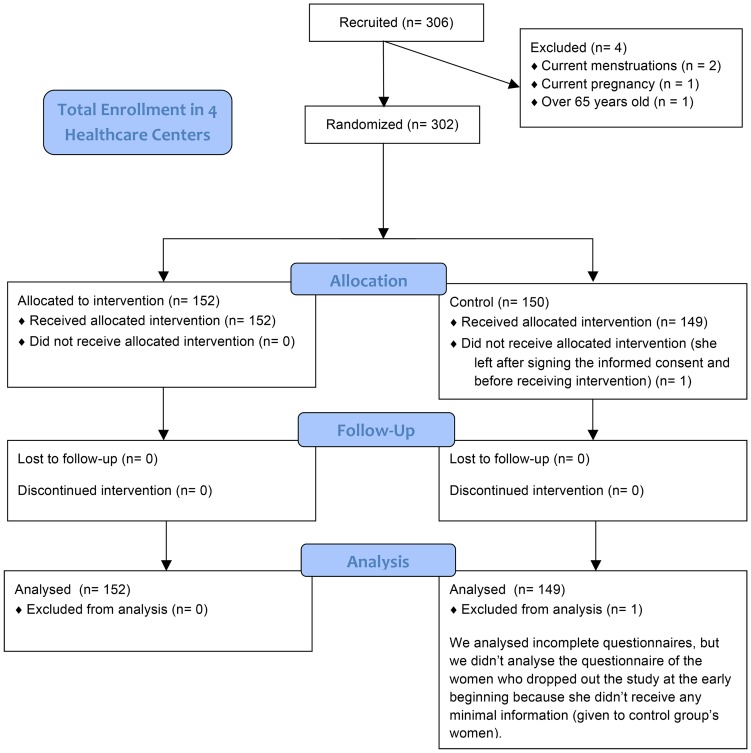
Study design and flow diagram. Flow diagram of patients in the study.

Participants were randomized in groups of 10 by order of arrival alternating into intervention or control group. We used subunits of 10 women instead of alternating one by one by order of arrival to avoid waiting time and diffusion of information given during the intervention in the waiting room (Figure 1).

### Intervention

“Basic information” was provided to all participants involving a discussion of approximately 5 minutes consisting of a brief explanation about the cervical screening campaign conducted by the University Center Hospital of Yaoundé and University Hospitals of Geneva and the use of Self-HPV as our method for cervical cancer screening. Explanations on how to perform the test were also given to all participants. The intervention group received in addition to the “basic information”, an “educational intervention” in the shape of a culturally-tailored video promoting a positive attitude towards cervical cancer screening. The video took 6 minutes to explain the role of HPV in cervical cancer and pre-cancer as well as the way to perform the Self-HPV test, its reliability and its relevancy as a screening test; see Video S1.

### Questionnaire

Primary outcome was the knowledge about cervical cancer. Secondary outcome was the acceptability and confidence/trust in the Self-HPV. To address these outcomes we designed a questionnaire consisting of 3 parts:

The first part assessed socio-demographic characteristics (age, and education level), sexual behavior (number of lifetime sexual partners) and gynecological history (history of abnormal cervical cytology, cervical therapy).

The second part assessed knowledge about cervical cancer, screening and HPV. This part consisted of 7 closed-ended questions with 3-point scaled answers (yes, no, don't know). Does HPV cause AIDS? (No), Does HPV cause Cervical Cancer? (Yes), Is HPV a Sexually Transmitted Disease? (Yes), Can men be infected with HPV? (Yes), Does a vaccination against HPV exist? (Yes), Can HPV induced lesions be treated? (Yes).

The third part evaluated Self-HPV acceptability by assessing embarrassment, pain, anxiety, discomfort, degree of relaxation and confidence. Responses were evaluated on a 4-point scale as follows: “not at all”, “slightly”, “moderately” and “very”.

The general willingness to perform Self-HPV was tested by evaluating the willingness to perform Self-HPV regularly and whether participants would recommend Self-HPV to friends and/or family. Furthermore we evaluated the preferred sampling side and the willingness to perform Self-HPV at home. We drafted this third part based on the methodology of previous studies [19], [20].

This questionnaire was addressed to all participants. All women answered part 1 of the questionnaire onto inclusion. The control group answered part 2 and 3 after performance of Self-HPV. The intervention group answered these two parts following the educational intervention and Self-HPV.

### Statistical treatment of data

All statistical analysis were performed using Microsoft Office Excel 2007 and OpenEpi: Open Source Epidemiologic Statistics for Public Health, Version 2.3.1. Mann-Whitney-U-test and chi-square test were used to analyze differences between intervention and control group. Tests were considered statistically significant when the *p*-value was <0.05.

The study was approved by the National Research Committee of Cameroon in early July 2012 for an recruiting period from July 24^th^ to August 8^th^ 2012 (approbation number: 159/CNE/SE/2012). The trial was registered with ISRCTN (registration number: ISRCTN78123709). The authors confirm that all ongoing and related trials for this intervention are registered.

## Results

### Participant's characteristics and settings

A total of 302 women were randomized of which 301 completed the full process and were included in the analysis. One subject was excluded because she did not answer the questionnaire. The randomization divided 152 women in the interventional group and 149 in the control group. The women were from Yaoundé or its surroundings with an average of 19 minutes walking distance from their home to the healthcare center (range 1–120 min). They were essentially French speakers (97%) and Christian (59.8% catholic and 23.9% protestant). The mean age was 38.3 years (range 25–65 years). Most of them were married (56.1%) and had children (88.0%) with a mean number of 3.6 deliveries. The mean age of first sexual intercourse was 17.7 years and mean number of sexual lifetime partners was 3.7. The majority of them were educated (56.5% high-school, 24.6% university). Twenty-five percent indicated having had at least one previous cervical cancer screening by a health-care professional (Table 1).

**Table 1:** Baseline socio-demographic characteristics of intervention and control participants (n = 301).

Characteristics	Intervention n = 152(%)		Control n = 149 (%)	
**Age**				
Median	38.5		38.2	
25–34	58	(38.2)	66	(44.3)
35–44	50	(32.9)	41	(27.5)
45–54	38	(25.0)	25	(16.8)
55–65	6	(3.9)	16	(10.7)
Unknown	0	(0.0)	1	(0.7)
**Marital status**				
Married and unmarried couples	89	(58.6)	82	(55.1)
Single	63	(41.4)	66	(44.2)
Unknown	0	(0.0)	1	(0.7)
**Education**				
No formal education and primary school	19	(12.5)	28	(18.8)
High School	94	(61.8)	76	(51.0)
University	37	(24.3)	37	(24.8)
Other and Unknown	2	(1.3)	8	(5.4)
**Profession**				
Housewife	27	(17.8)	49	(32.9)
Employee	74	(48.7)	44	(29.5)
Independent	27	(17.8)	32	(21.5)
Other and Unknown	24	(15.8)	24	(16.1)
**Age at first sexual intercourse** (mean)	17.7		17.8	
**Number of different sexual partners** (mean)	3.4		3.9	
**Number of deliveries** (mean)	3.4		3.8	
**Number of children per woman** (mean)	3.1		3.1	
**Previous screening**				
Yes	36	(23.7)	40	(26.9)
No	116	(76.3)	107	(71.8)
Unknown	0	(0.0)	2	(1.3)
**Time from house to healthcare center** (mean)	18.1 min.		20.1 min.	
**Spoken language** *				
French	147		145	
English	12		10	
**Religion**				
Catholic	86	(56.6)	94	(63.1)
Protestant	40	(26.3)	32	(21.5)
Pentecostal	10	(6.6)	6	(4.0)
Others and Unknown	16	(10.5)	17	(11.4)

*some women speak two or more languages.

### Knowledge and acceptability

Women in the intervention group showed significantly higher knowledge about HPV than women in the control group (p<0.05) (Table 2). Acceptability for each studied item was high in both groups (Table 3) and none of the items evaluating acceptability changed with better knowledge about HPV and cervical cancer. No significant difference of acceptability between women with good or poor knowledge was observed in the two groups. Most women would agree to repeat Self-HPV regularly (95.3%) and would recommend it to their family and/or friends (98.0%). Both groups named healthcare centers as their preferred place to perform Self-HPV (Table 4). Most women would like to receive more information about HPV and cervical cancer screening, without any statistical difference between intervention and control group (p = 0.2).

**Table 2:** Answers to questionnaire about HPV and cervical cancer, intervention versus control group.

	Intervention n = 152 (%)		Control n = 149 (%)		*P*-value*
**Have you ever heard about HPV?**					
Yes	73	(48.0)	59	(39.6)	<0.05**
No	77	(50.7)	71	(47.7)	
Doesn't know	2	(1.3)	17	(11.4)	
**Does HPV cause AIDS?**					
Correct answer	20	(13.2)	14	(9.4)	0.27
Wrong answer or don't know	126	(82.9)	133	(89.3)	
**Does HPV cause cervical cancer?**					
Correct answer	142	(93.4)	65	(43.6)	<0.05
Wrong answer or don't know	10	(6.6)	82	(55.1)	
**Is HPV a sexually transmitted disease?**					
Correct answer	142	(93.4)	36	(24.2)	<0.05
Wrong answer or don't know	8	(5.3)	107	(71.8)	
**Can men be infected by HPV?**					
Correct answer	137	(90.1)	34	(22.8)	<0.05
No or don't know	15	(9.9)	111	(74.5)	
**Does it exist any vaccination against HPV?**					
Correct answer	143	(94.1)	31	(20.8)	<0.05
Wrong or don't know	9	(5.9)	115	(77.1)	
**Can HPV lesions be treated?**					
Correct answer	119	(78.3)	66	(44.3)	<0.05
Wrong answer or don't know	30	(19.8)	79	(53.0)	
**Poor knowledge** **	5	(3.3)	101	(67.8)	<0.05
**Mediocre knowledge**	23	(15.1)	33	(22.1)	
**Good Knowledge**	124	(81.6)	15	(10.1)	

* Chi-square test and **Mann-Whitney-U-test was used to analyze the data.

** Poor knowledge: 0-2 right answers out of 6 questions, mediocre knowledge: 3–4 right answers out of 6 questions, good knowledge: 5–6 right answers out of 6 questions.

There were 11 missing answers in the intervention group and 21 missing answers in the control group caused by some women answering only part of the questionnaire. This is why the sum of answers for each item can differ and be less than the total of participants (301 women).

HPV (human papillomavirus), AIDS (Acquired Immunodeficiency Syndrome).

**Table 3:** Acceptability score for individual parameters for Self-HPV (mean scores on a scale of 1–4).

	Intervention n = 152 (%)		Control n = 149 (%)		*P*-value
**Embarassement**					
None	127	(83.6)	115	(77.2)	0.34
Low	16	(10.5)	21	(14.1)	
Moderate	4	(2.6)	7	(4.7)	
High	2	(1.3)	1	(0.7)	
No answer	3	(2.0)	5	(3.4)	
**Pain**					
None	131	(86.2)	128	(85.9)	0.99
Low	13	(8.6)	16	(10.7)	
Moderate	4	(2.6)	0	(0.0)	
High	1	(0.7)	1	(0.7)	
No answer	3	(2.0)	4	(2.7)	
**Anxiety**					
None	114	(75.0)	105	(70.5)	0.52
Low	25	(16.4)	31	(20.8)	
Moderate	7	(4.6)	5	(3.4)	
High	3	(2.0)	2	(1.3)	
No answer	3	(2.0)	6	(4.0)	
**Confidence**					
None	13	(8.6)	8	(5.4)	0.12
Low	32	(21.1)	21	(14.1)	
Moderate	24	(15.8)	25	(16.8)	
High	76	(50.0)	89	(59.7)	
No answer	7	(4.6)	6	(4.0)	
**Discomfort**					
None	130	(85.5)	126	(84.6)	0.84
Low	12	(7.9)	10	(6.7)	
Moderate	5	(3.3)	6	(4.0)	
High	4	(2.6)	1	(0.7)	
No answer	1	(0.7)	6	(4.0)	
**Relaxed**					
None	16	(10.5)	21	(14.1)	0.45
Low	37	(24.3)	21	(14.1)	
Moderate	14	(9.2)	16	(10.7)	
High	82	(53.9)	86	(57.7)	
No answer	3	(2.0)	5	(3.4)	
**Complexity**					
None	111	(73.0)	115	(77.2)	0.60
Low	21	(13.8)	13	(8.7)	
Moderate	2	(1.3)	6	(4.0)	
High	12	(7.9)	8	(5.4)	
No answer	6	(4.0)	7	(4.7)	

Mann-Whitney-U-test was used to analyze the data. The test was considered as statistically significant when p-value was <0.05.

**Table 4:** Willingness to perform HPV self-sampling (intervention vs control).

	Intervention n = 152 (%)		Control n = 149 (%)		P-value*
**Agree to do regularly Self-HPV**					
Yes	150	(98.7)	137	(92.0)	p = 0.31
No	2	(1.3)	2	(1.3)	
Don't know and unknown	0	(0.0)	10	(6.7)	
**Would recommend Self-HPV to friends/family**					
Yes	148	(97.4)	147	(98.7)	p = 0.85
No	3	(1.9)	0	(0.0)	
Don't know and unknown	1	(0.7)	2	(1.3)	
**Preferred place to perform self-sampling**					
Health Centre	111	(73.0)	120	(80.5)	p = 0.30
Home	26	(17.1)	14	(9.4)	
Doesn't know and no answer	13	(8.6)	15	(10.1)	
Other	2	(1.3)	0	(0.0)	

* *Mann-Whitney-U-test was used to analyze the data. p<0.05 was considered as significant.*

### Potential barriers to Self-HPV

A group of 17 women (5.6%) considered that they had religious beliefs, which were contradicting Self-HPV, however all of them performed Self-HPV voluntarily during the study. 5 women (1.7%) felt unable to judge whether their religion allowed Self-HPV and 276 (91.7%) confirmed that they had no religious belief against Self-HPV. Inquiring the need of their partner's approval to perform Self-HPV 52 women (17.3%) answered that they needed their partner's agreement, 8 women (2.7%) did not know or did not answer and 241 (80.1%) responded that they did not need their partner's agreement to perform Self-HPV.

### Willingness to perform Self-HPV

A total of 291 women (96.7%) would recommend Self-HPV to their friends or family (Table 4). Among the 44 expressed opinions, reasons to recommend Self-HPV were: *“To know the HPV status”* (n = 15), “Because Self-HPV is *easy to do*” (n = 7), *“quickly performed”* (n = 2) and *“because it is better to screen than to treat”* (n = 2).

## Discussion

Promoting cervical cancer screening in low resource settings like sub-Saharan African countries should be an important issue for health policy makers. To date, only a limited number of studies about educational interventions for cancer prevention were conducted in sub-Saharan Africa [15]–[18]. Studies with different outcome measures provided data on the effectiveness of interventions, however there still was limited evidence to support the fact that these educational interventions increased cancer knowledge or acceptability and uptake of screening tests [16]–[18]. Our current trial explored an educational method for cervical cancer prevention.

Our findings support that a culturally sensitive short video-based intervention influenced and improved knowledge about HPV and cervical cancer. These results were consistent with other studies where video interventions improve knowledge and uptake of available screening methods for colorectal cancer [21], breast cancer [22] and cervical cancer [23]. Previous reports demonstrated that appropriate educational interventions about breast cancer improved women's breast health knowledge, and also reduced their perceived barriers to early detection and screening [24]. This supports that cervical cancer health knowledge would be a necessary step for changing cervical health seeking behavior. The educational intervention in our study was effective in improving the actual knowledge level. However, although it would have been a desirable outcome in the context of public education, the improved awareness about HPV and cervical cancer screening among the studied population did not necessarily translate into a more positive participation and uptake of cervical cancer screening.

Our results indicated that Self-HPV was well accepted by participants and that most of them felt confident about their self-performance. They would also recommend the test to others or would do it again if they had the opportunity. Overall acceptability did not differ between the control and interventional group and analysis of subscale scores did not show that participants perceived Self-HPV differently or were less confident of their ability to perform it. We noticed that the educational intervention did not affect the willingness to perform Self-HPV probably because the participant's motivation was already high before the educational intervention [25].

Important determinants to be considered for Self-HPV implementation were religious believes and the entourage's approval, as they may be a barrier to the participation in screening [26]. We did not observe that Cameroonian women had any cultural values or religious beliefs, which were a barrier to Self-HPV participation. While exploring how women were supported by their husbands or partners before performing Self-HPV, we did not identify “family” factors limiting women's participation in cervical cancer screening.

The strength of this study was its large sample size, drawing participants from different areas of Yaoundé, and the use of validated measures for assessing acceptability for HPV self-sampling. One weakness was that acceptability may be overestimated as this study was voluntary-based. The study was conducted in healthcare centers, in which the women were seeking healthcare and where the availability of health workers was higher, thus providing more health information than in other settings. This more welcoming setting and the voluntary participation may limit the generalization of the high acceptability of Self-HPV detected in our study. Finally, as women agreed to perform Self-HPV as a condition of study participation, they may represent a group that had higher knowledge about cervical cancer screening as well as a higher Self-HPV acceptability than others. Notwithstanding the limitations of the study, an educational intervention did not improve knowledge, and Self-HPV could be pointed out as one of the main instrument for future cervical cancer prevention in low resource settings like Cameroon.

Finally, healthcare centers appeared to be a favorable place for Self-HPV. It was the same case in Uganda [13], where the lack of privacy at home was perceived as a barrier for the use of self-sampling. In Kenya, women clearly preferred to perform the test at home [27]. It would be interesting to understand the determinants of the choice of the preferred location for self-sampling. Therefore, further in-depth research would be needed to explore Cameroonian women's cervical cancer screening behavior, which could represent a basis for improving the national information and educational efforts.

In conclusion, a simple educational intervention can lead to significant improvement in health knowledge. No significant difference of acceptability to perform Self-HPV test was observed between women with good or poor knowledge as acceptability to perform Self-HPV was notably high in both groups.

Further information would be required in order to understand the relation between the improved knowledge and cervical cancer screening adherence.

## Supporting Information

Checklist S1 **CONSORT checklist.**
(DOCX)

Protocol S1 **Trial protocol in English.**
(DOCX)

Protocol S2 **Trial protocol in French.**
(DOCX)

Video S1 **Video with English subtitles.** A culturally-tailored video explaining the role of HPV in cervical cancer and pre-cancer as well as the way to perform the Self-HPV test, its reliability and its relevancy as a screening test. The video is available, in French with English subtitles.
(MP4)

## References

[1] Eddy DM (1990) Screening for Cervical Cancer. Ann Intern Med 113: 214–226. 2115753 10.7326/0003-4819-113-3-214

[2] Free K, Roberts S, Bourne R, Dickie G, Ward B, et al (1991) Cancer of the cervix–old and young, now and then. Gynecol Oncol 43: 129–136. 1743554 10.1016/0090-8258(91)90058-d

[3] Kohler Ba, Ward E, McCarthy BJ, Schymura MJ, Ries LaG, et al (2011) Annual report to the nation on the status of cancer, 1975–2007, featuring tumors of the brain and other nervous system. J Natl Cancer Inst 103: 714–736. 21454908 10.1093/jnci/djr077 PMC3086878

[4] Gichangi P, Estambale B, Bwayo J, Rogo K, Ojwang S, et al (2003) Knowledge and practice about cervical cancer and Pap smear testing among patients at Kenyatta National Hospital, Nairobi, Kenya. Int J Gynecol Cancer 13: 827–833. 14675320 10.1111/j.1525-1438.2003.13612.x

[5] Miller a B, Nazeer S, Fonn S, Brandup-Lukanow a, Rehman R, et al (2000) Report on consensus conference on cervical cancer screening and management. Int J Cancer 86: 440–447. 10760836 10.1002/(sici)1097-0215(20000501)86:3<440::aid-ijc22>3.0.co;2-a

[6] Franco E, Syrjänen K, Wolf C De, Syrjanen K, Ferenczy A, et al (1996) New developments in cervical cancer screening and prevention. Geneva, Switzerland, June 17–19 1996. Workshop. Cancer Epidemiol Biomarkers Prev 5: 853–856. 8896898

[7] Bosch FX, Lorincz A, Muñoz N, Meijer CJLM, Shah KV (2002) The causal relation between human papillomavirus and cervical cancer. 10.1136/jcp.55.4.244 PMC1769629 11919208

[8] Gaffikin L, Blumenthal PD, Emerson M, Limpaphayom K (2003) Safety, acceptability, and feasibility of a single-visit approach to cervical-cancer prevention in rural Thailand: a demonstration project. Lancet 361: 814–820. 12642047 10.1016/s0140-6736(03)12707-9

[9] Sankaranarayanan R, Gaffikin L, Jacob M, Sellors J, Robles S (2005) A critical assessment of screening methods for cervical neoplasia. Int J Gynaecol Obstet 89 Suppl 2: S4–S12. 15823266 10.1016/j.ijgo.2005.01.009

[10] Cuzick J, Arbyn M, Sankaranarayanan R, Tsu V, Ronco G, et al (2008) Overview of human papillomavirus-based and other novel options for cervical cancer screening in developed and developing countries. Vaccine 26 Suppl 1: K29–41. 18847555 10.1016/j.vaccine.2008.06.019

[11] Petignat P, Faltin DL, Bruchim I, Tramèr MR, Franco EL, et al (2007) Are self-collected samples comparable to physician-collected cervical specimens for human papillomavirus DNA testing? A systematic review and meta-analysis. Gynecol Oncol 105: 530–535. 17335880 10.1016/j.ygyno.2007.01.023

[12] Tebeu P-M, Major a L, Rapiti E, Petignat P, Bouchardy C, et al (2007) The attitude and knowledge of cervical cancer by Cameroonian women; a clinical survey conducted in Maroua, the capital of Far North Province of Cameroon. Int J Gynecol Cancer 18: 761–765. 17868337 10.1111/j.1525-1438.2007.01066.x

[13] Mitchell S, Ogilvie G, Steinberg M, Sekikubo M, Biryabarema C, et al (2011) Assessing women's willingness to collect their own cervical samples for HPV testing as part of the ASPIRE cervical cancer screening project in Uganda. Int J Gynaecol Obstet 114: 111–115. 21669428 10.1016/j.ijgo.2011.01.028

[14] Berner A, Hassel SB, Tebeu PM, Untiet S, Kengne-Fosso G, et al.. (2013) Human papillomavirus self-sampling in Cameroon: Women's uncertainties over the reliability of the method are barriers to acceptance. J Low Genit Tract Dis. 10.1097/LGT.0b013e31826b7b51 23422643

[15] Rwamugira J, Maree JE (2012) The findings of a nurse-lead intervention for detection and prevention of oral cancer. A pilot study. Eur J Cancer Care (Engl) 21: 266–273. 22111655 10.1111/j.1365-2354.2011.01310.x

[16] Wright KO, Kuyinu Ya, Faduyile Fa (2010) Community education on cervical cancer amongst market women in an urban area of Lagos, Nigeria. Asian Pac J Cancer Prev 11: 137–140. 20593944

[17] Nzarubara RG (1999) Control of breast cancer using health education. East Afr Med J 76: 661–663. 10734533

[18] Chirwa S, Mwanahamuntu M, Kapambwe S, Mkumba G, Stringer J, et al (2010) Myths and misconceptions about cervical cancer among Zambian women: rapid assessment by peer educators. Glob Health Promot 17: 47–50. 20595342 10.1177/1757975910363938 PMC4123628

[19] Dzuba IG, Diaz EY, Allen B, Leonard YF, Ponce ECL, et al (2002) The Acceptability of Self-Collected Samples for HPV Testing vs. the Pap Test as Alternatives in. J Womens Health Gend Based Med 11: 265–275. 11988136 10.1089/152460902753668466

[20] Waller J, McCaffery K, Forrest S, Szarewski a, Cadman L, et al (2006) Acceptability of unsupervised HPV self-sampling using written instructions. J Med Screen 13: 208–213. 17217611 10.1177/096914130601300409

[21] Gimeno-García AZ, Quintero E, Nicolás-Pérez D, Parra-Blanco A, Jiménez-Sosa A (2009) Impact of an educational video-based strategy on the behavior process associated with colorectal cancer screening: a randomized controlled study. Cancer Epidemiol 33: 216–222. 19747893 10.1016/j.canep.2009.08.003

[22] Wang JH, Schwartz MD, Brown RL, Maxwell AE, Lee MM, et al (2012) Results of a Randomized Controlled Trial Testing the Efficacy of a Culturally Targeted and a Generic Video on Mammography Screening among Chinese-American Immigrants. Cancer Epidemiol Biomarkers Prev 21: 1923–1932. 22971901 10.1158/1055-9965.EPI-12-0821 PMC3542829

[23] Love GD, Tanjasiri SP (2012) Using Entertainment-Education to Promote Cervical Cancer Screening in Thai Women. J Cancer Educ: 1–6. 10.1007/s13187-012-0369-5 PMC4372047 22581487

[24] Hall CP, Hall JD, Pfriemer JT, Wimberley PD, Jones CH (2010) Effects of a Culturally Sensitive Education Program and Beliefs of Hispanic Women. 34: 1195–1202. 10.1188/07.ONF.1195-1202 18024346

[25] Papa D, Moore Simas Ta, Reynolds M, Melnitsky H (2009) Assessing the role of education in women's knowledge and acceptance of adjunct high-risk human Papillomavirus testing for cervical cancer screening. J Low Genit Tract Dis 13: 66–71. 19387125 10.1097/LGT.0b013e31818a53f0

[26] Nene B, Jayant K, Arrossi S, Shastri S, Budukh A, et al.. (2007) Determinants of women' s participation in cervical cancer screening trial, Maharashtra, India. 031195. 10.2471/BLT.06.031195 PMC2636321 17546307

[27] Rositch AF, Gatuguta A, Choi RY, Guthrie BL, Mackelprang RD, et al (2012) Knowledge and acceptability of pap smears, self-sampling and HPV vaccination among adult women in Kenya. PLoS One 7: e40766. 22808257 10.1371/journal.pone.0040766 PMC3393696

