# Correction: Impact of an Educational Intervention on Women’s Knowledge and Acceptability of Human Papillomavirus Self-Sampling: A Randomized Controlled Trial in Cameroon

**DOI:** 10.1371/journal.pone.0117927

**Published:** 2015-02-06

**Authors:** The PLOS ONE Staff

The first two authors, Gaëtan Sossauer and Michel Zbinden, should be noted as contributing equally to this work.
