# Supplementary material for: Impact of an Educational Intervention on Women's Knowledge and Acceptability of Human Papillomavirus Self-Sampling: A Randomized Controlled Trial in Cameroon
Source: PLoS One. 2014 Oct 15;9(10):e109788. doi: 10.1371/journal.pone.0109788 (PMC4198132; doi:10.1371/journal.pone.0109788)
Supplement: Protocol S1 — Trial protocol in English. (DOCX) [file pone.0109788.s002.docx]

STUDY PROTOCOL

1. **Impact of an Educational Program Towards Women’s Awareness and Confidence in Human Papillomavirus Self-Sampling: A Randomized Controlled Trial in Cameroon**

**Main Investigators**

Sossauer, Gaëtan

Zbinden, Michel

**Co-investigators**

Tebeu, Pierre-Marie

Petignat, Patrick (Project Manager)

**Academic Affiliation**

Faculté de Médecine de Genève (Switzerland)

Faculté de Médecine Yaoundé (Cameroon)

**ABSTRACT**

**Context:** The development of new screening methods such as self-HPV sampling. It is necessary to assess women’s understanding of these methods in order to maximize their adherence to this type of screening.

**Objectives:** Assessing the impact of an educational program on knowledge and attitude towards self-HPV sampling.

**Material and methods:**Women aged 25 to 65 (target population for HPV screening in Cameroon) will be randomly selected for one of two groups : (i) self-sampling with no educational program (control group) or (ii) self-sampling preceded by an educational program (intervention group). Patients will be randomized within each healthcare center.

The control group will receive an instruction manual and a self-sampling kit.

The intervention group will go through an educational program in the form of a video presentation in the waiting room, and will receive an instruction manual.

The patient group that did not receive any educational program (control group) will be compared to the group that received an educational program (intervention group). Statistical analysis will assess participants’ confidence in the method in both groups. It is expected that 60% of patients in the control group and 80% in the intervention group will trust the HPV self-sampling screening method.

**Expected Results:**

The results should help better define the information to be given to women prior to the use of a self-sampling device for HPV screening.

## PROJECT IN DETAILS

**Introduction**

Cervical cancer is the second most common type of female cancer worldwide. It is responsible for 480,000 cancers and 280,000 deaths per year, of which approximately 87% occur in developing countries^[^^[[1]](#endnote-1)]^. In Cameroon, there are 1000 to 1200 new cases of cervical cancer annually, and it is the leading cause of cancer death for women. In addition, these women are often young women in charge of a family, and the impact on the family and social structure is important. The difference between the incidence of cervical cancer between Western countries and developing countries is mainly due to the absence of screening ^[^^[[2]](#endnote-2)]^.

The cervical cancer and precancerous lesions preceding the onset of cancer are caused by HPV (Human Papillomavirus) virus infection. HPV are a group of viruses that infect stratified squamous epithelia and mucosae causing abnormal cell proliferation resulting in the formation of warts and rarely of cancer. When the HPV infection persists, the subject is at risk of developing precancerous lesions and cancer. It takes 10 to 15 years for a persistent primary HPV infection to progress towards a cancer. This span of 10 to 15 years allows an effective prevention of cervical cancer through early diagnosis of precancerous lesions, and early treatment to stop further cancer development.

In countries with limited resources different detection techniques have been evaluated such as cytology, VIA (visual inspection with acetic acid), VILLI (visual inspection with Lugol) and more recently HPV test (detection of HPV DNA by PCR). There is currently more and more evidence that HPV testing will become the standard test for the screening for cervical cancer in developing countries.

Although current HPV tests are very effective, they have not been designed for use in countries with limited resources. However, a new generation of tests, simple, accurate, fast and culturally acceptable is being developed. These tests have the potential to reduce cervical cancer in countries with limited resources.

The HPV test requires a cervical or vaginal sample that may be taken by a qualified health provider. This sampling can also be done by women themselves. A self-performed vaginal swab is more likely to be accepted by women in many circles. It can be performed at home or at a healthcare center and does not require a gynecological examination with a speculum. Cervical or vaginal swabs can be stored and transported to a laboratory to be analyzed by qualified personnel.

When implementing the prevention of HPV through screening, it is wise to evaluate the population’s awareness of the link between cervical cancer and HPV, as well as the acceptability of women's self-sampling. Effective information could help overcome cultural barriers and increase adherence to screening.

**State of Knowledge**

Several studies have evaluated women’s preference between self-sampling and cervical cytology performed by a gynecologist ^[^^[[3]](#endnote-3)]^. The majority of women prefer self-sampling, regardless of ethnic or religious affiliations. However, it appears that women with a higher level of education and higher income feel more comfortable performing a self-sampling^[^^[[4]](#endnote-4)]^. Some causes of reluctance to practice self-sampling have been observed, such as cost, lack of confidence in medicine and the fear of not being able to understand the instructions ^[^^[[5]](#endnote-5)]^.

Preliminary research (to be published shortly) conducted in Cameroon as part of a collaboration between the University Hospital of Yaoundé (CHUY), the National Committee for the Fight against Cancer and University Hospitals of Geneva has shown that an education program could have a significant impact on the level of understanding, the acceptance of and confidence in the screening method. This study suggests that in the absence of any educational program, women do not trust self-sampling tests, and prefer a test performed by a gynecologist during a gynecological examination ^[^^[[6]](#endnote-6)]^.

The feasibility and convenience of self-sampling for the detection of cervical cancer have been clearly demonstrated (to be published shortly). For a country like Cameroon, with few physicians, the benefit of self-sampling is evident in terms of medical resources and cost. However, the implementation of a program based on self-sampling must be understood by women (HPV knowledge), must be viewed as culturally acceptable and must be accompanied by an awareness of the benefits of the screening method. Once trust is established and information is accurate and communicated effectively, and once women’s questions are satisfied, barriers will be removed, which will ensure better adhesion to cervical cancer screening.

**Objectives**

Assessing the impact of an educational program on knowledge and attitude towards self-HPV sampling.

**Population and methods**

Education will be provided through a video (same information for all), so that it can be reproduced later if proven effective.

**Control group « leaflet only »:**

- Each participant is recruited in the waiting room.
- The study is explained and consent is obtained.
- An instruction manual is given.
- Each participant answers questions on knowledge about HPV and cervical cancer (part 1 and 2 of the questionnaire).
- Each participant performs self-sampling.
- Each participant then completes questions about the method’s acceptability and attitude about performing self-HPV sampling (part 3 of the questionnaire).

*NB* : for ethical reasons, after the above steps are completed, the leaflet-only group will receive information in the form of a video about cervical cancer and HPV.

**Intervention group « leaflet and video counseling »:**

- Each participant is recruited in the waiting room.
- The study is explained and consent is obtained.
- An instruction manual is given.
- Information video on the link between cervical cancer and HPV and on the validity of self-sampling as a screening method is watched.
- Each participant performs self-sampling.
- Each participant then completes questions about method’s acceptability and attitude about performing HPV self-sampling (part 3 of the questionnaire).

**Statistical Analysis**

We will undertake an individual randomization. A randomized controlled trial is the reference method for assessing the impact of any health program. It assesses the impact of this program, using a direct comparison with a group that does not go through the program.

The final outcome measured is confidence in the method.

The expected level of acceptability of the method is up to 60% in the control group and 80% in the intervention group. So we need to recruit a minimum of 182 patients to demonstrate a 20% difference between the two groups, knowing that a statistical power of 80% is desired (α = 0.05; β = 0.20).

**Potential biases**

The study aims at evaluating an intervention (informational) to improve adherence of patients to a new screening test (HPV self-sampling). Using individual randomization, three biases are encountered.

First, a risk of "contamination" between the two groups exists. People in the control group may hear of the educational program before entering the study, due to discussions outside the specific context of the study. Especially because we count on word-of-mouth to recruit new participants.

Second, a risk of making different recommendations to each group or participant exists and is common when information is given orally. We try to avoid this bias by using a video for the intervention group and no video for the control group in order to be sure everyone receives the same information.

Third, the null hypothesis in a randomized, single unit randomization test, states that different participants are independent. This assumption is violated when we try to assess a program supposed to improve quality of care. Indeed, patients followed by the same physician or in the same hospital have a higher probability of receiving the same care or of responding the same way to the program compared to patients treated by a different physician or in different hospitals. To avoid this bias, we will recruit participants in different healthcare centers.

Another potential bias, unrelated to the statistical method, is that women will be recruited in waiting rooms. Therefore, they are not necessarily representative of the entire eligible population for screening, because they are already seeking care.

**Expected benefits**

The lack of confidence in medicine and the fear of not being able to understand instructions make HPV self-sampling a questionable solution for its use in countries with limited resources. HPV screening and vaccination are the main methods of prevention of cervical cancer. But they only protect concerned women, i.e. those who are already aware of the potential risk of HPV virus infection. Prevention should be added to HPV screening and vaccination programs.

This study expects to demonstrate a statistically significant improvement of approximately 20% in the understanding, compliance and trust in the HPV self-sampling method.

**Bibliography**

1. ^[]^ HPV and cervical cancer in the 2007 report. Vaccine 2007;25 Suppl 3:C1–230. [↑](#endnote-ref-1)
2. ^[]^ US Census [homepage on the Internet] Washington, D.C.: Demographic Indicators. Country Summary: Cameroon; 2007. <http://www.census.gov/aboutus/stat_int.html> [cited 2008 June 23] [↑](#endnote-ref-2)
3. ^[]^ Mitchell S, Ogilvie G, Steinberg M, Sekikubo M, Biryabarema C, Money D. Assessing women’s willingness to collect their own cervical samples for HPV testing as part of the ASPIRE cervical cancer screening project in Uganda. Int J Gynaecol Obstet 2011;114(2):111–5. [↑](#endnote-ref-3)
4. ^[]^ Forrest S, McCaffery K, Waller J, et al. Attitudes to self-sampling for HPV among Indian, Pakistani, African-Caribbean and white British women in Manchester, UK. J Med Screen 2004;11(2):85–8. [↑](#endnote-ref-4)
5. ^[]^  Waller J, McCaffery K, Forrest S, Szarewski A, Austin J, Wardle J. Acceptability of unsupervised HPV self-sampling using written instructions. J Med Screen 2006;13(4):208–13. [↑](#endnote-ref-5)
6. ^[]^ McCarey C, Pirek D, Tebeu PM, Boulvain M, Doh AS, Petignat P. Awareness of HPV and cervical cancer prevention among Cameroonian healthcare workers. BMC Women’s Health 2011;11:45 [↑](#endnote-ref-6)
