# Supplementary material for: Impact of an Educational Intervention on Women's Knowledge and Acceptability of Human Papillomavirus Self-Sampling: A Randomized Controlled Trial in Cameroon
Source: PLoS One. 2014 Oct 15;9(10):e109788. doi: 10.1371/journal.pone.0109788 (PMC4198132; doi:10.1371/journal.pone.0109788)
Supplement: Protocol S2 — Trial protocol in French. (DOCX) [file pone.0109788.s003.docx]

PROTOCOLE D’ETUDE

1. **Impact d’une intervention éducative sur les connaissances et attitudes envers l’auto-prélèvement HPV au Cameroun : un essai randomisé**

**Investigateurs principaux**

Sossauer Gaëtan

Zbinden Michel

**Co-investigateurs**

Pierre-Marie Tebeu

Patrick Petignat (responsable du master)

**Affiliations**

Faculté de Médecine de Genève (Suisse)

Faculté de Médecine Yaoundé (Cameroun)

**RESUME**

**Contexte :** Le développement de nouvelles méthodes de dépistage telles que l’auto-prélèvement HPV et sa compréhension par le public doit être évalué afin d’optimiser l’adhésion des femmes à la méthode.

**Objectifs :** Evaluer l’impact d’une action éducative sur les connaissances et l’attitude envers l’auto-prélèvement HPV.

**Matériel et méthodes :**Les femmes âgées entre 25 et 65 ans (population cible pour le dépistage au Cameroun) subiront une randomisation individuelle pour (i) un auto-prélèvement sans action éducative (groupe contrôle) ou (ii) un auto-prélèvement précédé d’une action éducative (groupe intervention). Les patientes seront randomisées au sein de chaque unité de soin.

Le groupe contrôle recevra un mode d’emploi et un set d’auto-prélèvement.

Le groupe intervention recevra une action éducative sous forme d’une présentation vidéo en salle d’attente, ainsi qu’un mode d’emploi.

Le groupe de patiente n’ayant pas reçu d’intervention éducative (groupe contrôle) sera comparé au groupe ayant reçu une intervention éducative (groupe intervention). L’analyse statistique évaluera la confiance en la méthode dans les deux groupes. On présume que 60% des patientes du groupe contrôle et 80% du groupe intervention auront confiance en la méthode.

**Résultats attendus :**

Les résultats devraient permettre de mieux définir les axes à privilégier en termes d’information aux femmes avant la mise en route d’un dépistage de type auto-prélèvement HPV.

## PROJET DÉTAILLÉ

**Introduction**

Le cancer du col utérin représente la seconde cause de cancer féminin dans le monde. Il est responsable de 480’000 cancers et 280’000 décès par année, dont environ 87% dans les pays en voie de développement ^[^^[[1]](#endnote-1)]^. Au Cameroun, on compte annuellement entre 1'000 et 1'200 nouveau cas de cancer du col et il représente la première cause de décès par cancer chez la femme. De plus, les femmes atteintes sont souvent des femmes jeunes en charge de famille et l’impact sur la structure familiale et sociale est important. La différence entre l'incidence des cancers du col entre les pays occidentaux et les pays à faibles ressources s'explique essentiellement par l'absence de dépistage ^[^^[[2]](#endnote-2)]^.

Le cancer du col utérin et les lésions précancéreuses qui précèdent l’apparition d’un cancer ont pour origine une infection par un virus, le papillomavirus humain (HPV). Les HPV constituent un groupe de virus qui infectent les épithéliums squameux stratifiés et les muqueuses provoquant une prolifération cellulaire anormale aboutissant à la formation de verrue et rarement d’un cancer. Lorsque l’infection HPV persiste, alors le sujet est à risque de développer des lésions précancéreuses et un cancer. Cette période entre primo-infection HPV et développement d’un cancer prend entre 10 et 15 ans, ce qui permet une prévention efficace du cancer du col par un diagnostic précoce des lésions précancéreuses et l’instauration d’un traitement évitant le développement en cancer.

Différentes techniques de dépistage ont été évaluées dans les pays à faibles ressources, tels que la cytologie, la VIA (inspection visuelle après application d’acide acétique), la VILLI (inspection visuel après application de lugol) et plus récemment le test HPV. Il y a actuellement de plus en plus d’évidence comme quoi le test HPV deviendra le test de référence pour le dépistage du cancer du col dans les pays en voie de développement.

Bien que les tests HPV actuels soit très efficace, ils n’ont pas été conçu pour une utilisation dans des environnements à faibles ressources. Toutefois, une nouvelle génération de test, simple, précis, rapide et acceptable est en train d’être développée. Ces derniers ont le potentiel de réduire le cancer du col dans les pays à faibles ressources.

Le test HPV peut être effectué par un prélèvement cervical ou vaginal effectué par un prestataire de soins qualifié. Ce prélèvement peut également être effectué par les femmes elles-mêmes. L’auto-prélèvement vaginal s’avère être acceptable pour les femmes dans de nombreux milieux. Il peut être réalisé à domicile ou dans un centre de soin et ne nécessite pas d’examen gynécologique au spéculum. Les prélèvements cervicaux ou vaginaux peuvent être stockés et transportés vers un laboratoire pour être analysés par du personnel qualifié.

Dans la perspective d'une mise en place d’une prévention secondaire (dépistage) basée sur l’identification du HPV, il est judicieux d'évaluer d’une part les connaissances de la population concernant l’association entre le cancer du col et le HPV et ensuite l’acceptabilité des femmes pour l’auto-prélèvement. Une bonne information pourrait permettre de dépasser les barrières culturelles et d’augmenter l’adhésion au dépistage.

**Etat des connaissances sur le sujet**

Plusieurs études ont évaluées la préférence des femmes entre l’auto-prélèvement et la cytologie cervicale réalisée par le gynécologue ^[^^[[3]](#endnote-3)]^. La majorité des femmes préfèrent l’auto-prélèvement et ceci indépendamment des appartenances ethniques ou religieuse. Il semble toutefois que les femmes ayant un meilleur niveau d’éducation et un niveau de revenu plus haut se sentent plus à l’aise pour réaliser l’auto-prélèvement ^[^^[[4]](#endnote-4)]^. Certaines réticences ou freins à l’auto-prélèvement ont été observé, ce sont les coûts, le manque de confiance en la médecine et la peur de ne pas réussir à comprendre les instructions ^[^^[[5]](#endnote-5)]^.

Des travaux préliminaires conduits au Cameroun dans le cadre d’une collaboration entre le Centre Hospitalier Universitaire de Yaoundé (CHUY), le Comité National de Lutte contre le Cancer et les Hôpitaux Universitaires de Genève ont montré qu’une action éducative pourrait avoir un impact important dans la compréhension, l’acceptabilité et la confiance en la méthode par les femmes dépistées (travaux en cours de publication). Ces travaux suggèrent qu’en l’absence d’action éducative, les femmes n’ont pas confiance au test et préfèrent un examen gynécologique avec un test réalisé par un médecin gynécologue ^[^^[[6]](#endnote-6)]^.

La faisabilité et la facilité de l’auto-prélèvement pour le dépistage du cancer du col utérin à clairement été démontré (travaux en cours de publication). Pour un pays tel que le Cameroun, qui manque de médecin, le bénéfice d’un auto-prélèvement est évident en terme de ressource médicale et de coût. Toutefois, la mise en route d’un programme basé sur l’auto-prélèvement doit être compris par les femmes (connaissance du HPV), doit être acceptable et doit être lié à une confiance dans les bénéfices que peut apporter ce dépistage. Si une relation de confiance s’instaure, que la communication et l’information est bonne et que les femmes sont satisfaites, certaines barrières seront levées et permettront d’obtenir l’adhésion de la population cible au dépistage du cancer du col utérin.

**Objectifs**

Evaluer l’impact d’une action éducative sur les connaissances et l’attitude envers l’auto-prélèvement

**Population et méthode**

L'intervention effectuée sera sous forme de film vidéo (même information pour tous), afin qu’elle puisse être reproduite ultérieurement si celle-ci s'avère efficace.

**Groupe contrôle « leaflet only »:**

- La participante est recrutée dans la salle d'attente.
- L’étude lui est expliquée et le consentement est obtenu.
- Un mode d’emploi est transmis.
- Elle complète les questions portant sur les connaissances du HPV et du cancer du col (partie 1 et 2 du questionnaire).
- Elle effectue l’auto-prélèvement
- La patiente est ensuite invitée à compléter les questions sur l’acceptabilité de la méthode et l’attitude à effectuer l'auto-prélèvement HPV (partie 3 du questionnaire).

*Note* : par souci d’éthique, le groupe *leaflet only* reçoit, une fois les étapes ci-dessus effectuées, les informations sous forme d’une vidéo à propos de l’association entre le cancer du col et le HPV.

**Groupe intervention « leaflet and video conselling »:**

- La participante est recrutée dans la salle d'attente.
- L’étude est expliquée et le consentement obtenu.
- Un mode d’emploi est transmis.
- Des informations sous forme d’une vidéo sur l’association entre le cancer du col et le HPV, ainsi que la validité de l’auto-prélèvement comme méthode de dépistage sont données.
- Un auto-prélèvement est effectué.
- La patiente est ensuite invitée à compléter les questions sur les connaissances du HPV et du cancer du col ainsi que sur l’acceptabilité et l’attitude à effectuer l'auto-prélèvement HPV.

**Analyse statistique**

Nous allons procéder à une randomisation individuelle. L’essai contrôlé randomisé représente la méthode de référence pour évaluer l'impact d'une intervention en santé. Il permet d'évaluer l'impact de cette intervention, à l'aide d'une comparaison directe avec un groupe qui ne reçoit pas l'intervention.

L’issue finale évaluée sera la confiance en la méthode.

L’effet attendu de l’intervention est de 60% dans le bras contrôle et environ 80% dans le bras intervention. On peut calculer qu’il faut au minium recruter 182 patientes pour mettre en évidence une différence de 20% entre le groupe contrôle et le groupe intervention, sachant que l’on désir obtenir une puissance statistique de 80% (α=0,05 ; β=0,20).

**Biais potentiel**

L’étude vise à évaluer une intervention d’amélioration de l’adhésion (information) des patientes à un nouveau test de dépistage (auto-prélèvement HPV). En utilisant la méthode de randomisation individuelle on rencontre trois biais.

Premièrement, le problème majeur de cet essai est le risque de « contamination » entre les groupes. En effet, le groupe contrôle peut être informé de l’intervention éducationnelle, lié aux échanges entre patientes en dehors du cadre spécifique de l’étude.

Deuxièmement, il est impossible d’imaginer que nous allons effectuer une recommandation à un groupe, puis que nous allons oublier cette recommandation pour l’autre groupe. Nous essayons de contourner ce biais par l’utilisation d’une vidéo pour le groupe intervention et pas de vidéo pour le groupe contrôle.

Troisièmement, l'hypothèse de base dans un essai randomisé à unité de randomisation individuelle est que les différents malades sont indépendants. Cette hypothèse est violée dans la mise en œuvre d’interventions destinées à améliorer la qualité des soins. En effet, les malades suivis par un même médecin ou dans un même hôpital (à qui l’intervention est destinée) ont une probabilité plus élevée d'avoir la même prise en charge ou de répondre de la même manière à l’intervention, que des malades traités par des médecins différents ou dans des hôpitaux différents. Donc pour contourner ce biais, nous passerons dans plusieurs centre de santé.

Un autre biais potentiel indépendant de la méthode statistique est le fait que les femmes seront recrutées dans les salles d’attentes et par conséquent ne sont pas nécessairement représentatives de l’ensemble de la population féminine éligible pour le dépistage (elles sont déjà dans un processus de recherche de soin).

**Bénéfices attendus**

Le manque de confiance en la médecine et la peur de ne pas réussir à comprendre les instructions font de l’autotest HPV une solution douteuse quant à son utilisation dans les pays à faible ressources. Le dépistage et la vaccination HPV sont les principaux facteurs de prévention contre le cancer du col de l’utérus. Mais ils protègent seulement les femmes concernées, c’est-à-dire sensibilisée par le risque potentiel du virus HPV. La prévention est devrait être ajoutée à ce mix de stratégies (dépistage et/ou vaccination).

Nous attendons donc dans notre étude une amélioration statistiquement significative d’environ 20% dans la compréhension, la compliance et la confiance envers cet autotest HPV.

**Bibliographie**

1. ^[]^ HPV and cervical cancer in the 2007 report. Vaccine 2007;25 Suppl 3:C1–230. [↑](#endnote-ref-1)
2. ^[]^ US Census [homepage on the Internet] Washington,D.C.: Demographic Indicators. Country Summary:Cameroon; 2007. <http://www.census.gov/aboutus/stat_int.html> [cited 2008 June 23] [↑](#endnote-ref-2)
3. ^[]^ Mitchell S, Ogilvie G, Steinberg M, Sekikubo M, Biryabarema C, Money D. Assessing women’s willingness to collect their own cervical samples for HPV testing as part of the ASPIRE cervical cancer screening project in Uganda. Int J Gynaecol Obstet 2011;114(2):111–5. [↑](#endnote-ref-3)
4. ^[]^ Forrest S, McCaffery K, Waller J, et al. Attitudes to self-sampling for HPV among Indian, Pakistani, African-Caribbean and white British women in Manchester, UK. J Med Screen 2004;11(2):85–8. [↑](#endnote-ref-4)
5. ^[]^  Waller J, McCaffery K, Forrest S, Szarewski A, Austin J, Wardle J. Acceptability of unsupervised HPV self-sampling using written instructions. J Med Screen 2006;13(4):208–13. [↑](#endnote-ref-5)
6. ^[]^ McCarey C, Pirek D, Tebeu PM, Boulvain M, Doh AS, Petignat P. Awareness of HPV and cervical cancer prevention among Cameroonian healthcare workers. BMC Womens Health 2011;11:45 [↑](#endnote-ref-6)
